# Salpingectomy may decrease antral follicle count but not live birth rate for IVF-ET patients aged 35–39 years: a retrospective study

**DOI:** 10.1186/s13048-020-00678-9

**Published:** 2020-07-20

**Authors:** Tong Chen, Feiyan Zhao, Qin Wang, Chang Liu, Yonglian Lan, Shuyu Wang, Zhimin Xin, Xiaokui Yang

**Affiliations:** Department of Human Reproductive Medicine, Beijing Obstetrics and Gynecology Hospital, Capital Medical University, 251 Yao jia yuan Road, Chaoyang District, Beijing, 100026 China

**Keywords:** In vitro fertilization-embryo transfer, Antral follicle count, Live birth, Salpingectomy

## Abstract

**Purpose:**

Problems with fallopian tubes are one of the main reasons for women to undergo in vitro fertilization-embryo transfer (IVF-ET). A large proportion of women with ectopic pregnancy, fallopian tube obstruction and hydrosalpinx have had one or both fallopian tubes removed by salpingectomy. With increasing age, ovarian reserve deteriorates, the numbers of retrieved oocytes, available embryos and high-quality embryos are reduced, and the live birth rate for women treated with IVF treatment is affected. Thus, it is important to understand how salpingectomy affects live birth rates for IVF patients of different ages. This study analyzed how patients’ age and salpingectomy influenced ovarian reserve, ovarian response and pregnancy outcomes for infertile women undergoing IVF-ET.

**Methods:**

A total of 1922 patients that underwent IVF-ET treatment from January 1, 2012, to December 31, 2018, were included in this retrospective study. The patients were divided into two groups according to whether or not they had a previous history of salpingectomy. The salpingectomy (group A, 534 patients) and control groups (group B, 1388 patients) were then further divided into two subgroups according to patient age (age<35 years, and age 35–39 years). Ovarian reserve, ovarian response, and IVF outcomes were investigated for each subgroup. Logistic regression model was used to estimate the relationship between clinical pregnancy and live births and patients’ baseline characteristics.

**Results:**

In the salpingectomy group, antral follicle counts (AFC) were significantly lower for the subgroup aged 35 to 39 years compared with the control group. But this difference did not appear in women younger than 35 years. In addition, there were no significant differences in levels of basal follicle stimulation hormone (FSH), basal luteinizing hormone (LH), basal estradiol (E_2_), total gonadotropins (Gn) dose, duration of Gn, numbers of retrieved oocytes, fertilization rates, numbers of available embryos, live birth rates, clinical pregnancy rates, miscarriage rates, ectopic pregnancy rates, or multiple pregnancy rates between the salpingectomy group and the control group (*P* > 0.05). Age is a risk factor for the clinical pregnancy and live birth.

**Conclusion:**

Salpingectomy may decrease antral follicle count but not live birth rate for IVF-ET patients aged 35–39 years. The increased female age was negative related with clinical pregnancy and live birth.

## Introduction

Infertility is estimated to affect 8–12% of couples of child-bearing age worldwide [1]. However, with the rapid development of assisted reproductive technology, an increasing number of infertile women may still achieve a successful pregnancy through the process of in vitro fertilization-embryo transfer (IVF-ET). Infertility may be caused by ovulation disorders, fallopian tube issues, endometriosis, male disorders, and other factors [2]. Tubal infertility caused by fallopian tube obstruction, hydrosalpinx, tubal ligation, or salpingectomy accounts for 25–35% of female infertility [3]. Many women underwent salpingectomy (removal of one or both fallopian tubes) due to ectopic pregnancy, hydrosalpinx or fallopian tube obstruction. According to the guideline issued by The American College of Obstetricians and Gynecologists (ACOG), salpingectomy is a routine therapy for ectopic pregnancy (EP) cases when the patient is exhibiting signs of intraperitoneal bleeding or ongoing pelvic pain, or when patients have contraindications to more conservative medical treatments [4]. The American Society for Reproductive Medicine (ASRM) also recommends salpingectomy as a therapy for patients with extensive dense peritubal adhesions, a surgically irreparable hydrosalpinx, or a fallopian tube that is damaged beyond repair by infection or endometriosis [5]. IVF was first used to treat cases of tubal factor infertility, and tubal infertility is one of the chief indications for IVF.

The effect of salpingectomy on ovarian reserve and ovarian response in women who underwent IVF treatment is still a controversial issue. Some studies have found that basal follicle-stimulating hormone (bFSH) levels were significantly increased, and serum anti-Müllerian hormone (AMH) levels were decreased in women who had a salpingectomy compared to women with tubal factor infertility but no salpingectomy [6, 7]. Pereira et al. suggested that IVF-ET patients who had a previous unilateral salpingectomy required higher gonadotropin (Gn) doses and longer stimulation duration when undergoing a controlled ovarian stimulation (COS) compared to patients who had not undergone salpingectomy [8]. However, some investigators showed that there was no significant influence on ovarian reserve, ovarian response, or the number of retrieved oocytes before and after salpingectomy [9–16]. With advancing female age, declines in number and quality of oocytes are the primary reasons for decreasing IVF outcomes, especially in terms of live birth rates [17]. The effect of female age and previous salpingectomy history on live birth rates during the IVF cycle is the main focus of this investigation.

Some recently published studies demonstrated that the clinical pregnancy rate for women undergoing IVF after salpingectomy was comparable to that for women with no previous salpingectomy [8, 9, 16, 18]. We investigated whether salpingectomy affected the ovarian reserve, ovarian response and pregnancy outcomes for infertile women of different ages undergoing IVF. In our present study, 1922 women who underwent IVF treatment within a five-year period were included and divided into the salpingectomy group and control group as described above. We found that bFSH levels, basal luteinizing hormone (bLH) levels, basal estradiol (bE_2_) levels, antral follicle counts (AFC), total Gn dose, and duration of relatively the same for both groups. There also were no significant differences between the two groups based on live birth rates, clinical pregnancy rates, spontaneous abortion rates, ectopic pregnancy rates and multiple pregnancy rates. Within the salpingectomy group, AFCs were significantly lower for the age 35–39 years patient group (group A2) compared to the control group (group B2).

## Materials and methods

### Study population

This retrospective cohort study included patients receiving IVF treatment in the Department of Human Reproductive Medicine, Beijing Obstetrics and Gynecology Hospital, Capital Medical University during the period from January 1, 2012 to December 31, 2018. A total of 1922 patients were recruited for this study. For all patients, controlled ovarian stimulation data is included from the first IVF cycle only. This study was approved by the ethics committee of Beijing Obstetrics and Gynecology Hospital, Capital Medical University.

Patients were divided into salpingectomy group and control group according to whether they had salpingectomy before. In each group, patients were then divided into two subgroups according to age (less than 35 and 35–39 years).

Inclusion criteria were age <40 years, infertility duration > 12 months and regular menstrual cycle (21–35 days). For the salpingectomy group, patients were chosen if they had undergone unilateral or bilateral salpingectomy to treat tubal pregnancy or hydrosalpinx and the interval time between salpingectomy and IVF was 1–3 years. For the control group, patients were selected if they were diagnosed with unilateral or bilateral fallopian tube obstruction but had not undergone salpingectomy.

Patients diagnosed with endometriosis, adenomyosis, polycystic ovarian syndrome, uterine fibroids, genital organ deformity, autoimmune disease, metabolic disorders, infertility caused by male factors, or who had been treated with steroids or immune inhibitors in the 6 months prior to IVF were excluded from the study.

### IVF treatment

Patients were treated according to one of four controlled ovarian stimulation protocols based on age and ovarian reserve: GnRH-agonist long protocol (GnRH agonist administration in the luteal phase of the previous cycle), prolonged GnRH-agonist protocol (prolonged GnRH-agonist administration in Day 2–3 of the previous cycle), GnRH-agonist short protocol (daily GnRH agonist administration starting with menstrual day 1–2 of the IVF cycle), GnRH antagonist protocol (daily GnRH antagonist administration from Day 6–7).

Exogenous Gn (human menopausal gonadotrophin, 75 IU, Livzon Pharmaceutical Group, China; or Menopur, 75 IU, Ferring GmbH, Germany; or Gonal-F, 75 IU, Merck Serono, Germany) were administered at a dose ranging from 150 to 375 IU/d based on patients’ age, BMI and ovarian reserve. Patients had serial transvaginal ultrasound scans and serum LH, E_2_ and P were measured until the follicles reached maturity. The dosage of Gn was adjusted depending on the ovarian response.

Human chorionic gonadotrophin (hCG) (250 μg, Merck Serono Inc., Geneva, Switzerland) was subcutaneously injected at night when the maximum follicle reached 18 mm or at least two follicles reached 17 mm in diameter based on transvaginal ultrasound. Oocyte retrieval was conducted by transvaginal ultrasound-guided follicular puncture 36–38 h after hCG trigger.

All patients received embryo transfer after oocytes were retrieved. Embryo transfer was performed on day 2 or day 3 after oocyte retrieval. The luteal phase was supported by daily oral progesterone (Progesterone capsules, 100 mg, bid, Xianju Pharma, China and Progesterone soft capsules, 0.2 g, tid, Besins Manufacturing Belgium, France or Progesterone sustained-release vaginal gel, 90 mg, qd, Merck Serono, Germany) starting on the day of oocyte retrieval. Pregnancy was diagnosed using a positive serum hCG test 14–16 days after embryo transfer and clinical pregnancy was confirmed if the gestational sac could be observed by transabdominal ultrasound 28–35 days after embryo transfer. Progesterone support was maintained until the 10th week of pregnancy.

### Outcome measure

We analyzed number of retrieved oocytes, fertilization rate, clinical pregnancy rate, spontaneous abortion rate, ectopic pregnancy rate, live birth rate, and multiple pregnancy rate. Live birth was defined as one or more newborns with vital signs after 28 completed weeks of gestation. Ectopic pregnancy was defined as a pregnancy occurring outside the uterine cavity. Multiple pregnancy was defined as more than one fetus for a pregnancy. The main measure of interest was live birth rate (per cycle and per transfer). Live birth was defined as one or more babies showing signs of life after 28 weeks of gestation.

### Statistical analysis

SPSS 23.0 software (IBM Corporation, New York, America) was used for data analyses. In the comparison of groups and subgroups (group A vs. group B, group A1 vs. group A2, group B1 vs. group B2, group A1 vs. group B1, group A2 vs. group B2), statistical differences for non-normally distributed continuous variables were evaluated using Mann–Whitney U-test. Categorical variables and rates were evaluated using chi-square test and Fisher’s exact test. Logistic regression analysis was performed for clinical pregnancy and live birth including the following covariates: age, BMI, infertility duration, basal hormone levels, and antral follicle count. Two-sided *P* values < 0.05 were considered statistically significant. Results are presented as mean ± standard deviation.

## Results

### Patients’ baseline characteristics

A total of 1922 patients in their first IVF cycle were recruited for the present study. Patients who had previous history of salpingectomy due to ectopic pregnancy or hydrosalpinx were divided into the salpingectomy group (group A, 534 patients), and patients receiving IVF treatment due to fallopian tube obstruction were included in the control group (group B, 1388 patients). The patients’ baseline characteristics are summarized in Table 1. All patients were similar in age, infertility duration, BMI, basal FSH levels, basal LH levels, basal E_2_ levels and AFC. No significant differences were found between the patients in the two groups (*P* > 0.05).

**Table 1:** Basic characteristics of patients in salpingectomy group (A) and control group (B)

	Group A (*n* = 534)	Group B (*n* = 1388)	*P* value
Age (years)	32.18 ± 3.81	32.01 ± 3.75	NS
Infertility duration (years)	3.69 ± 2.64	3.44 ± 2.09	NS
BMI (kg/m^2^)	23.06 ± 3.47	22.87 ± 3.50	NS
Type of infertility			NS
Primary infertility	326 (61.0%)	834 (60.1%)	
Secondary infertility	208 (39.0%)	554 (39.9%)	
Basal FSH (IU/L)	7.61 ± 2.56	7.76 ± 2.72	NS
Basal LH (IU/L)	3.86 ± 1.91	4.05 ± 2.13	NS
Basal E_2_ (pg/ml)	44.84 ± 21.44	47.30 ± 31.22	NS
Antral follicle counts	8.11 ± 4.22	8.18 ± 3.55	NS

*Abbreviations*: *FSH* follicle-stimulating hormone, *LH* luteinizing hormone, *E*_*2*_ estradiol

Statistical differences were calculated using the Mann–Whitney U-test, except for “type of infertility,” for which the chi-square test was used

Results are significantly different if *P* < 0.05. Values are expressed as mean ± standard deviation unless indicated otherwise (Type of infertility is expressed as the number of IVF cycles)

### Controlled ovarian stimulation parameters and IVF outcomes

Table 2 shows a summary of the different COS parameters and pregnancy outcomes for the salpingectomy group and control group. Durations and total dosages of Gn, endometrium thicknesses and hormone levels on hCG day, numbers of oocytes retrieved, fertilization rates, numbers of available embryos, and pregnancy outcomes were not significantly different between patients in the salpingectomy and control groups (*P* > 0.05).

**Table 2:** Controlled ovarian stimulation conditions and pregnancy outcomes for patients in salpingectomy group (A) and control group (B)

	Group A (n = 534)	Group B (n = 1388)	*P* value
Gonadotrophin duration (days)	10.40 ± 2.31	10.23 ± 2.29	NS
Gonadotrophin dosage (IU)	2468.78 ± 866.33	2419.39 ± 866.31	NS
Endometrial thickness on hCG day (mm)	10.99 ± 5.43	10.77 ± 3.62	NS
LH on hCG day (IU/L)	2.42 ± 2.39	2.23 ± 2.17	NS
E_2_ on hCG day (IU/L)	2664.79 ± 1676.78	2783.86 ± 1765.68	NS
P on hCG day (pg/ml)	1.26 ± 0.84	1.05 ± 0.64	NS
Number of oocytes retrieved	9.47 ± 4.65	9.35 ± 3.93	NS
Number of fertilized oocytes	7.19 ± 3.78	7.30 ± 3.39	NS
Oocyte fertilization rate (%)	78.84 ± 15.77	78.25 ± 15.67	NS
Number of transferred embryos	1.90 ± 0.30	1.90 ± 0.31	NS
Number of available embryos	5.06 ± 2.75	5.16 ± 2.67	NS
Clinical pregnancy rate (%)	40.6 (217)	41.7 (579)	NS
Spontaneous abortion rate (%)	15.7 (34)	15.0 (87)	NS
Live birth rate (%)	32.0 (171)	33.2 (462)	NS
Ectopic pregnancy rate (%)	5.5 (12)	5.2 (30)	NS
Multiple pregnancy rate (%)	15.2 (33)	15.9 (92)	NS

Statistical differences were calculated using the Mann–Whitney U-test. Values are expressed as mean ± standard deviation unless indicated otherwise

Clinical pregnancy rate, Spontaneous abortion rate, Live birth rate, Ectopic pregnancy rate, and Multiple pregnancy rate were calculated using the chi-square test or Fisher’s exact test

Results are significantly different at *P* < 0.05

### Ovarian response markers and pregnancy outcomes for different subgroups

In order to clarify whether salpingectomy affects ovarian response and live birth rates for infertile women of different ages, patients in the salpingectomy and control groups were each divided into two subgroups according to age: age less than 35 (salpingectomy group: 376 patients, namely group A1; control group: 995 patients, namely group B1) and age 35–39 years (salpingectomy group: 158 patients, namely group A2; control group: 393 patients, namely group B2). Table 3 shows the ovarian response markers and pregnancy outcomes in each subgroup. No statistical differences were found between bFSH and bE_2_ levels, clinical pregnancy rate, spontaneous abortion rate, live birth rate, ectopic pregnancy rate or multiple pregnancy rate of any two subgroups (group A1 vs. group A2, group B1 vs. group B2, group A1 vs. group B1, group A2 vs. group B2, *P* > 0.05).

**Table 3:** Ovarian reserve markers and pregnancy outcomes in patients of different ages within salpingectomy group and control group

	Salpingectomy group		Control group	
	Group A1 (age <35 years, *n* = 376)	Group A2 (age 35–39 years, *n* = 158)	Group B1 (age <35 years, *n* = 995)	Group B2 (age 35–39 years, *n* = 393)
Basal FSH (IU/L)	7.57 ± 2.56	7.72 ± 2.57	7.62 ± 2.70	7.78 ± 2.77
Basal E_2_ (pg/ml)	45.52 ± 22.23	43.20 ± 19.36	46.37 ± 28.26	49.68 ± 37.68
Antral follicle counts	8.69 ± 4.35	6.75 ± 3.56^a,b^	8.25 ± 3.48	8.00 ± 3.71
Clinical pregnancy rate (%)	41.2 (155)	39.2 (62)	41.9 (417)	41.2 (162)
Spontaneous abortion rate (%)	14.8 (23)	17.7 (11)	13.9 (58)	17.9 (29)
Live birth rate (%)	33.0 (124)	29.7 (47)	34.0 (338)	31.6 (124)
Ectopic pregnancy rate (%)	5.2 (8)	6.5 (4)	5.0 (21)	5.6 (9)
Multiple pregnancy rate (%)	15.5 (24)	14.5 (9)	15.8 (66)	16.0 (26)

*Abbreviations*: *FSH* follicle-stimulating hormone, *E*_*2*_ estradiol. Values are expressed as mean ± standard deviation unless indicated otherwise

Statistical differences were calculated using the Mann–Whitney U-test

Clinical pregnancy rate, spontaneous abortion rate, live birth rate, ectopic pregnancy rate, and multiple pregnancy rate were calculated using the chi-square test or Fisher’s exact test

Results are significantly different if *P* < 0.05

^a^*P*<0.05 (vs. group A1)

^b^*P*<0.05 (vs. group B2)

In the salpingectomy group, AFCs were significantly lower for the 35–39 year age subgroup compared to the control group (group A2 vs. group B2, *P* < 0.05). However, this trend was not found in patients < 35 years between salpingectomy group and control group (group A1 vs. group B1, *P* > 0.05). We also found that AFCs were significantly lower in group A2 (35–39 year age subgroup) compared to the group A1 (< 35 year age subgroup, *P* < 0.05). Nevertheless, this change was not found between < 35 year age subgroup and 35–39 year age subgroup in patients who had not undergone salpingectomy (group B1 vs. group B2, *P* > 0.05).

In the same age subgroups, patients with a history of salpingectomy had lower clinical pregnancy rates and live birth rates and higher miscarriage rates compared to the control group, but this difference is not statistically significant (group A1 vs. group B1, group A2 vs. group B2, *P* > 0.05). The clinical pregnancy rate and live birth rate were lower and the miscarriage rate was higher with increasing age both in the salpingectomy group and the control group (group A1 vs. group A2, group B1 vs. group B2). Nevertheless, there were no significant differences among the subgroups (*P* > 0.05).

### Logistic regression of pregnancy outcomes in patients after IVF treatment

The logistic regression model (Table 4) showed that the age was the independent predictor of clinical pregnancy (OR = 0.972, 95% CI 0.946–0.998) and live birth (OR = 0.970, 95% CI 0.944–0.996) (*P* < 0.05). Other baseline characteristics such as BMI, infertility duration, basal hormone levels, and antral follicle count were not associated with pregnancy outcomes in the model.

**Table 4:** Logistic regression of pregnancy outcomes in patients after IVF treatment

Baseline parameter	B	SE (b)	Wald χ2	*P* value	ORs	95% CI	
						Lower	Upper
Clinical pregnancy							
Age	−0.034	0.013	6.546	0.033	0.972	0.946	0.998
BMI	−0.026	0.021	1.506	0.113	0.966	0.925	1.008
Infertility duration	−0.022	0.013	2.607	0.346	0.987	0.961	1.014
Basal FSH	−0.001	0.018	0.002	0.693	1.007	0.972	1.044
Basal LH	0.026	0.023	1.243	0.273	1.026	0.980	1.075
Basal E_2_	−0.001	0.002	0.209	0.765	1.000	0.996	1.003
AFC	0.016	0.013	1.688	0.143	1.019	0.994	1.045
Live birth							
Age	−0.030	0.014	4.951	0.026	0.970	0.944	0.996
BMI	−0.059	0.047	1.611	0.204	0.942	0.860	1.033
Infertility duration	0.039	0.032	1.530	0.216	1.040	0.977	1.106
Basal FSH	0.042	0.045	0.865	0.352	1.043	0.954	1.140
Basal LH	−0.014	0.051	0.075	0.784	0.986	0.892	1.090
Basal E_2_	0.001	0.004	0.162	0.687	1.001	0.994	1.008
AFC	0.016	0.028	0.329	0.566	1.016	0.962	1.074

*Abbreviations*: *B* regression coefficient, *SE (b)* standard errors of regression coefficient, *OR* odds ratio

The modified Hosmer-Lemshow goodness of fit χ2 test statistics were 8.551 (*P* = 0.382) and 10.380 (*P* = 0.239), respectively

## Discussion

IVF-ET has become the primary method for women with a history of salpingectomy to achieve a successful clinical pregnancy and live birth. In present study, we found that salpingectomy may decrease AFCs but not affect live birth rate for infertile women aged 35–39 years. Moreover, age is a risk factor for the clinical pregnancy and live birth.

Ovarian reserve is an indicator of the number and quality of follicles in the ovaries and is frequently assessed during IVF treatment with AFC and serum markers such as FSH, E_2_, AMH, or inhibin-B [19, 20]. Based on previous study, the effects of salpingectomy on ovarian reserve and response remains unclear. Dar et al. compared the duration and quantity of Gn administered during IVF treatment, and clinical pregnancy rate in 26 patients who underwent unilateral salpingectomy. There was no significant difference between any of the measured parameters [15]. A retrospective study reported that salpingectomy did not influence the ovarian response in 36 women who had a previous salpingectomy to treat hydrosalpinx or ectopic pregnancy [21]. In another retrospective analysis of 94 women with tubal factor infertility who underwent IVF-ET, there were no differences in ovarian response between women who had a previous salpingectomy compared with women who were diagnosed with tubal-factor infertility and had not undergone salpingectomy [10]. However, one published retrospective study that investigated 198 women who underwent unilateral or bilateral salpingectomy versus an infertility group without salpingectomy showed that the serum AMH was lower, and the FSH was significantly higher in women without salpingectomy compared with those with bilateral salpingectomy [7]. Chan et al. compared ovarian volume, AFC, and 3D power Doppler indices in 32 patients who underwent unilateral salpingectomy due to ectopic pregnancy. AFC and 3D power Doppler indices have a significant decrease on the operated side compared with the non-operated side in the laparoscopy group [22]. These results suggested that ovarian reserve may be affected by salpingectomy. Our study showed that there were no statistical differences between the bFSH levels, bLH levels, bE_2_ levels, AFC, or total dosage and duration of Gn of patients who had a history of salpingectomy and the control group. Methodological variations and sample sizes in studies may have accounted for differences in results.

It is well known that ovarian reserve is closely related to a woman’s age. To further explore how age and salpingectomy affect women who were undergoing IVF treatment, we divided women with or without salpingectomy into two subgroups according to age (age < 35 years and 35–39 years). We investigated the differences in ovarian reserve markers for women of different ages who had undergone the salpingectomy. The findings of our study show that women 35–39 years of age with salpingectomy history had significantly decreased AFCs. However, the live birth rate after IVF treatment did not change significantly compared to women without salpingectomy of the same age. There was no significant of ovarian reserve indicators and pregnancy outcomes in patients < 35 years old with and without salpingectomy history. A prospective cross-sectional study included 381 women aged 20–50 years with regular menstrual cycle and count number of AFC based on transvaginal ultrasound in the early follicular phase. The result showed that there were no significant differences in AFC among different age groups under 35 years, whereas a significant decrease after 35 years [23]. Bishop et al. analyzed the relationship between live birth and ovarian reserve in 9489 cycles among 8214 patients aged 21–44 years. The younger diminished ovarian reserve patients (age < 35 years old) indicated by bFSH and AFC, have the same live birth rate to normal ovarian reserve patients. Likewise, diminished ovarian reserve has poor correlation with oocyte quality for patients in this age group [24]. The results of the present study support the hypothesis that younger women may have partial compensation for declining ovarian reserve after salpingectomy, whereas that effect decreases with increased of female age. However, there were no statistical differences in live birth rates (the main parameter of interest) and other IVF outcomes, such as fertilization rates, clinical pregnancy rates, spontaneous abortion rates, ectopic pregnancy rates and multiple pregnancy rates between the salpingectomy group and the control group, regardless of the age of the patient.

It was also found that regardless of whether the patient had undergone salpingectomy or not, the live birth and clinical pregnancy rates of younger patients were higher than those of older patients, but there were no significant differences among the subgroups. Age is one of the strongest predictors of success for IVF treatment [25]. With increasing age, the decreased number of follicles and the reduced quality of oocytes are the reasons for increased spontaneous abortion rates and decreased clinical pregnancy and live birth rates. The reduced oocyte quality is usually due to abnormalities of the spindle in the oocyte nucleus, an increase in the incidence of aneuploidy, a decrease in the proliferation, and an increase in the apoptotic of granulosa cells [26]. Moreover, age-related reductions in ovarian reserve and oocyte quality may be due to decreased androgen levels [27], oxidative stress [28], dysfunctional cohesins [29], shortening of telomeres [30], and impaired mitochondrial metabolic activity [31].

We have noticed some deficiencies in our study. First, patients were not subgrouped according to unilateral or bilateral salpingectomy, which may affect overall outcomes. However, some studies have shown that unilateral or bilateral salpingectomy does not affect ovarian reserve function and ovarian response [32, 33]. Second, we did not analysis the effect of different underlying indications for salpingectomy on ovarian reserve, ovarian response, and pregnancy outcome. However, a study suggested that there were no significant differences in number of oocytes retrieved, oocyte fertilization rates, and clinical pregnancy rates for patients with different salpingectomy indications [8]. Finally, we measured basal hormone levels and AFC to assess ovarian reserve, but did not measure AMH levels. A well-designed randomized controlled trial with a large sample size may overcome the drawbacks of retrospective analysis to further elucidate whether salpingectomy affects ovarian reserves, ovarian responses and IVF outcomes.

## Conclusions

Our investigation suggests that in women aged 35 to 39 years, salpingectomy may significantly decrease AFC, which may indicate declined ovarian reserve. However, live birth rates as a result of IVF treatment did not differ significantly between patients 35–39 years old and those < 35 years old. Moreover, bFSH, bLH and bE2 levels, total Gn doses, duration of Gn, fertilization rates, numbers of available embryos, and other pregnancy outcomes were similar between the salpingectomy group and the control group, regardless of the age of the patient. The increased female age was negative related with clinical pregnancy and live birth. The increased female age was significantly negative associated with clinical pregnancy and live birth. To ensure more comprehensive analysis, further studies should use a larger sample size and include all relevant data.

## Data Availability

All data analyzed in this study are included as tables in this article.
